# Thermo-Responsive Gel Containing Hydroxytyrosol-Chitosan Nanoparticles (Hyt@tgel) Counteracts the Increase of Osteoarthritis Biomarkers in Human Chondrocytes

**DOI:** 10.3390/antiox11061210

**Published:** 2022-06-20

**Authors:** Anna Valentino, Raffaele Conte, Ilenia De Luca, Francesca Di Cristo, Gianfranco Peluso, Michela Bosetti, Anna Calarco

**Affiliations:** 1 Research Institute on Terrestrial Ecosystems (IRET)—CNR, Via Pietro Castellino 111, 80131 Naples, Italy; anna.valentino@iret.cnr.it (A.V.); ilenia.deluca@iret.cnr.it (I.D.L.); gianfranco.peluso@unicamillus.org (G.P.); 2 AMES Group Polydiagnostic Center, Via Padre Carmine Fico, 24, 80013 Casalnuovo di Napoli, Italy; raffaele.conte86@tiscali.it; 3 Elleva Pharma s.r.l., Via P. Castellino 111, 80131 Naples, Italy; francesca.dicristo@ellevapharma.com; 4 UniCamillus, International Medical University, 00131 Rome, Italy; 5 Dipartimento di Scienze del Farmaco, Università del Piemonte Orientale “A. Avogadro”, Largo Donegani, 2, 28100 Novara, Italy

**Keywords:** hydroxytyrosol-chitosan nanoparticles, injectable hydrogel, anti-inflammatory, anti-oxidative, osteoarthritis

## Abstract

Although osteoarthritis (OA) is a chronic inflammatory degenerative disease affecting millions of people worldwide, the current therapies are limited to palliative care and do not eliminate the necessity of surgical intervention in the most severe cases. Several dietary and nutraceutical factors, such as hydroxytyrosol (Hyt), have demonstrated beneficial effects in the prevention or treatment of OA both in vitro and in animal models. However, the therapeutic application of Hyt is limited due to its poor bioavailability following oral administration. In the present study, a localized drug delivery platform containing a combination of Hyt-loading chitosan nanoparticles (Hyt-NPs) and in situ forming hydrogel have been developed to obtain the benefits of both hydrogels and nanoparticles. This thermosensitive formulation, based on Pluronic F-127 (F-127), hyaluronic acid (HA) and Hyt-NPs (called Hyt@tgel) presents the unique ability to be injected in a minimally invasive way into a target region as a freely flowing solution at room temperature forming a gel at body temperature. The Hyt@tgel system showed reduced oxidative and inflammatory effects in the chondrocyte cellular model as well as a reduction in senescent cells after induction with H_2_O_2_. In addition, Hyt@tgel influenced chondrocytes gene expression under pathological state maintaining their metabolic activity and limiting the expression of critical OA-related genes in human chondrocytes treated with stressors promoting OA-like features. Hence, it can be concluded that the formulated hydrogel injection could be proposed for the efficient and sustained Hyt delivery for OA treatment. The next step would be the extraction of “added-value” bioactive polyphenols from by-products of the olive industry, in order to develop a green delivery system able not only to enhance the human wellbeing but also to promote a sustainable environment.

## 1. Introduction

Osteoarthritis (OA) is a chronic inflammatory degenerative disease affecting millions of people worldwide. OA leads to cartilage deterioration, inflammation of the synovial membrane, and subchondral bone sclerosis due to abnormal bone remodeling caused by an overproduction of enzymes degrading the extracellular matrix [1,2,3]. This disease considerably reduces the quality of life for patients and is associated with pain, transient morning stiffness, and crepitus felt in a joint on moving it [4,5,6]. A large body of evidence supports the involvement of inflammation and reactive oxygen species (ROS) production by chondrocytes in OA cartilage [7,8,9].

To date, numerous pharmacological and non-pharmacological therapies have been developed for the management of OA. However, the current therapies are limited to palliative care and do not exclude the necessity of surgical intervention [10,11]. Under alternative or adjuvant therapeutic schemes, regular dietary intake of natural functional foods containing polyphenols and other phytochemicals such as fruits, vegetables, whole grains, legumes, and olive oil [12,13,14,15,16,17] has been associated to a protective role in chronic disease prevention such as OA because of their anti-inflammatory and antioxidant properties [18].

Among all, Hydroxytyrosol (Hyt), a polyphenol found mainly in olive oil and raw olives, exerts strong antioxidant activity (as a potent radical scavenger and metals chelator) and acts as an anti-inflammatory as well as antithrombotic, antitumor, antimicrobial, and neuroprotective agent [19,20,21,22].

Fucelli et al. demonstrated the ability of Hyt to reduce inflammatory markers, such as Cyclooxygenase-2 (COX2) and Tumor Necrosis Factor alfa (TNF-α) and reduces oxidative stress on a mouse model of systemic inflammation [23]. Pre-treatment of Balb/c mice with Hyt (40 and 80 mg/Kg b.w.) prevented all lipopolysaccharide-induced effects and decreased oxidative stress. In another study, Cetrullo et al. demonstrated that Hyt inhibits the inflammatory response in vascular endothelial cells, macrophages, and monocytes [24]. Furthermore, Hyt reduces oxidative stress and damage, exerts pro-survival and anti-apoptotic actions, and favorably influences the expression of critical OA-related genes in human chondrocytes treated with stressors promoting OA-like features [25].

However, the amount of Hyt obtained through its natural sources’ consumption is considerably lower than the recommended daily intake able to exert its claimed health-promoting properties [26]. Furthermore, the local therapeutic concentration of Hyt following oral administration is limited due to its poor bioavailability and enzyme degradation. Encapsulation of Hyt could be a functional alternative strategy to preserve its the biological activity and to ensure controlled release of the latter increasing the residence time inside the joint. Chitosan biopolymer has been extensively used as a matrix for the encapsulation of a wide range of natural products due to its beneficial properties including biodegradability, biocompatibility, and low cost [27]. Moreover, the ionic gelation method allows to obtain drug-loaded chitosan nanoparticles with a controlled size and satisfactory encapsulation capacity, protecting polyphenols from enzymatic oxidation or degradation. However, polymeric particles present some significant limitations such as initial burst release, escape from the joint’s cavity, and in vivo rejection.

To overcome the above-mentioned drawbacks, in the present work, a localized drug delivery platform containing a combination of Hyt-loading chitosan nanoparticles (Hyt-NPs) and in situ forming hydrogel have been developed to derive the benefits of both hydrogels and nanoparticles. This thermo-sensitive formulation, based on Pluronic F-127 (F-127), hyaluronic acid (HA), and Hyt-NPs (called Hyt@tgel) presents the unique ability to be injected in a minimally invasive way into a target region as a freely flowing solution. When the temperature rises near the body temperature of 37 °C, hydrogel has in situ sol-to-gel transition accommodating the shape to the geometry of the treated area. 

HA, a naturally polysaccharide, represents one of the largest components of the extracellular matrix of articular cartilage and plays an important endogenous role in the protection of articular cartilage decreasing the gene expression of inflammatory cytokines. Moreover, HA degrade ECM enzymes with stimulating in vitro chondrocytes proliferation, and chondrogenesis by directing mesenchymal stromal cells (MSCs) differentiation and increasing type 2 collagen production [28,29,30]. Although HA represents a conventional treatment in knee OA management, several lines of clinical evidence have questioned the effectiveness of such therapies due to HA prompt in vivo degradation mediated by hyaluronidases and oxidative stress [31,32]. To prolong HA residence time and confer optimized product functionality, Pluronic F-127 (F-127) consisting of hydrophilic poly (ethylene oxide) (PEO) and hydrophobic poly (propylene oxide) (PPO) (PEO-PPO-PEO) was added. Moreover, reported by Young-seok Jung and colleagues [33], the addition of high-molecular-weight HA (Mw: ~1000 kDa) increases the mechanical strength of thermos-responsive hydrogel hindering the interactions between water and poloxamer molecules due to HA-assisted inter-micellar packing. Starting from the results obtained in the work of Young-seok Jung, nanocomposite hydrogel formulations (Hyt@tgels) were optimized to ensure gelation around 37 °C, as well as allowing Hyt release exerting antioxidant and anti-inflammatory activity on an in vitro induced inflammatory environment mimicking OA.

Based on the above, the developed platform may serve as both a Hyt delivery system and as a tissue engineering scaffold to stimulate the regeneration of a lesioned tissue and to prevent chondrocytes senescence providing an alternative and potentially more effective loco-regional approach to manage OA.

## 2. Materials and Methods

### 2.1. Materials

Hyt (>98% purity), chitosan medium molecular weight (50,000–190,000 Da, 75–85% deacetylated, viscosity < 200 mPa.s, 1% in acetic acid), lactic acid (DL-Lactic acid, powder), sodium tripolyphosphate (TPP, technical grade), Pluronic F-127, and Fluorescein isothiocyanate (FITC), 3,3′,5,5′-Tetramethylbenzidine (TMB), Thiobarbitoric acid (TBA), Dichloro-dihydro-fluorescein diacetate (DCFH-DA) and Ultrapure HA in the form of sodium hyaluronate medium molecular weight were purchased from Sigma Aldrich (Milan, Italy) and used as received. All other reagents used in the experiment were of analytical grade and when not indicated were purchased from Sigma Aldrich (Milan, Italy). 

### 2.2. Preparation and Physico-Chemical Characterization of Hyt-Loading Nanoparticles (Hyt-NPs)

A series of three Hyt-loading nanoparticles (Hyt-NPs) with varying chitosan concentration (0.1%, 0.5%, and 1% *w*/*w*) were obtained, with slight modifications, according to the well-known ionotropic gelation method [34]. Briefly, chitosan solution in 1% (*v*/*v*) lactic acid was prepared and stirred overnight at room temperature. TPP (5 mg/mL) and Hyt (10 mg) were dissolved in double distilled water to achieve different CS:TPP mass ratios. All solutions were filtered using 0.45 µm pore size membrane filters. TPP/Hyt solution was added dropwise into the chitosan solution under magnetic stirring (750 rpm) until a translucent Hyt-NPs suspension was formed. The suspension was stirred for 1 h at 1000 rpm at 25 °C to allow complete interaction. Then, the solution containing nanoparticles was ultrasonicated for 5 min at 40 kHz. Finally, Hyt-NPs were collected by cooling centrifugation (Frontiers 5718R, OHAUS, Milan, Italy) at 15,000 rpm for 45 min at 4 °C and washed with deionized water. FITC-loaded NPs were obtained by adding hydrophilic fluorescent probe into the TPP aqueous phase instead of Hyt, and the NPs prepared as described previously. Blank NPs were produced as negative control.

Particle Size (hydrodynamic diameter), polydispersity index (PDI), and zeta potential measurements were carried out on freshly prepared samples as reported in Conte et al. [35]. All samples were diluted in deionized water and measured at 25 °C using a Malvern Zetasizer (Malvern Instruments Ltd., Malvern, UK). The reported data are an average value of three measurements of the same sample. The particle size was confirmed by NanoSight NS300 Nanoparticles Tracking Analysis (NTA, Malvern Instruments, Amesbury, United Kingdom, UK). The fresh nanoparticle dispersions were centrifuged at 14,000 rpm for 30 min (5718R, OHAUS, Nänikon, Switzerland). The amount of drug entrapped in NPs was determined in triplicate indirectly by analyzing the amount of free Hyt in supernatant. The free Hyt in supernatant was quantified as described in Section 2.4.4 paragraph.

The encapsulation efficiencies of a series of Hyt-loaded nanoparticles were determined based on the following equation:

$$\mathrm{Encapsulation}\;\mathrm{Efficiency}\;\left(\mathrm{EE}\;\%\right)=\frac{\mathrm{Total}\;\mathrm{amount}\;\mathrm{of}\;\mathrm{Hyt}\;\mathrm{loaded}-\mathrm{Free}\;\mathrm{Hyt}\;\mathrm{in}\;\mathrm{supernatant}}{\mathrm{Total}\;\mathrm{amount}\;\mathrm{of}\;\mathrm{Hyt}\;\mathrm{loaded}}\times 100$$

### 2.3. Hyt-Loaded Hydrogel (Hyt@tgel) Preparation

Injectable hydrogels were prepared according to the cold method as reported by [33]. Briefly, HA (100 mg) and Pluronic F-127 concentrations of 12–25% (*w*/*v*, 5 mL) were mixed in double distilled water at a temperature below 4 °C to form hydrogels. The polymer solution was left for at least 24 h to ensure the complete dissolution. Meanwhile, lyophilized Hyt-NPs (1, 5, and 10 mg) were added to the polymer dispersion and stirred for 1 h at 4 °C. 

### 2.4. Hyt@tgel Characterization

#### 2.4.1. Gelation Time and Syringeability

The sol-gel phase transition (gelation time) of Hyt@tgels was determined by modified test tube inversion method [36]. An aliquot (1.0 mL) of each sample was prepared in a glass tube and then placed in a low temperature digital water bath. The solution was heated at the rate of 0.5 °C/min and after each minute the glass vial rotated 90° to check the gelling of the sample. Tsol-gel was determined as the temperature at which the gel did not exhibit gravitational flow during a period of 2 min when the tube was reversed. Averages and standard deviations of each sample were determined in triplicate.

Syringeability was assayed after injection of Hyt@tgel through a syringe with a 30-gauge needle. The solutions which were easily passed from the syringe were termed as pass and the solutions which were difficult to pass were termed as fail.

#### 2.4.2. Mechanical Strength Test

The mechanical strength of Hyt@tgels was measured in relation to its viscosity with a Brookfield viscometer (RVDV-II + P, Brookfield, WI, USA) set at 200 rpm router speed with increasing temperatures (20–65 °C, Equilibrium time: 1 °C/2 min (>35 °C) or 5 °C/10 min (<35 °C)).

#### 2.4.3. Short-Term Stability Studies

The physico-chemical stability of Hyt@tgels was determined upon 14-day storage at different temperatures (4.0 ± 0.5 °C and 25.0 ± 0.5 °C). At predetermined times, aliquots were centrifuged (12,000× *g*, 4 h, 20 °C) to separate nanoparticles from the hydrogel. All samples were analyzed for particle size, PDI, and % drug entrapment efficiency, and the results were compared with the initial values. The Hyt stability during storage was confirmed by HPLC analysis as described in Section 2.4.4 paragraph. 

#### 2.4.4. In Vitro Hyt Release

The cumulative Hyt release from the hydrogel formulations was determined using the dialysis bag method in phosphate buffer saline (PBS, pH 7.4). The Hyt@tgels formulations (1 mL) were sealed in pre-swollen cellulose membrane dialysis bags (3.5–5.0 kDa cut-off, Spectrum) and immersed into 5 mL of PBS buffer (pH 7.4) in a water bath at 37 °C shaken at 100 rpm for 5 days. At set time intervals, 5 mL of the release media was collected for Hyt analysis and replaced with the same volume of fresh PBS to maintain the sink conditions. Hyt released in the PBS media from the hydrogels was measured with liquid chromatography–tandem mass spectrometry (LC-MS/MS) as reported by [37]. The LC-MS/MS system consisted of a Shimadzu NexeraXR UHPLC (Shimadzu Italy, Milan, Italy) coupled to an LCMS 8060 turbo spray ionization triple-quadrupole mass spectrometer (LCMS 8060, Shimadzu Italy, Milan, Italy). The whole system was controlled by Lab Solution software. Separation of analytes was achieved using a 2.6 μm Kinetex polar C18 column (Phenomenex, Torrance, CA, USA). The mobile phase included Buffer A (0.1% formic acid in water) and Buffer B (0.1% formic acid in acetonitrile) in isocratic flow. The total run time was 7 min for each injection. The mass spectrometer was operated in the turbo-spray mode with negative ion detection. The detection and quantification of Hyt was accomplished by multiple reaction monitoring (MRM) with the transitions *m*/*z* 153.05 → 123.0 (quantifier); 153.05 → 93.0 (qualifier). The instrumental parameters tuned to maximize the MRM signals were nebulizing gas flow 3 L/min, heating gas flow 10 L/min, interface temperature 370 °C, DL temperature 250 °C, heat block temperature 450 °C, and drying gas flow 10 L/min.

### 2.5. In Vitro Cell Studies

#### 2.5.1. Cell Culture and Treatment

Human chondrocyte cells line C20A4 was obtained from American Type Culture Collection (ATCC, Manassas, VA, USA). It was maintained at 37 °C in a humidified atmosphere containing 5% CO_2_ in Dulbecco’s modified Eagle’s Medium/Nutrient Mixture F-12 (DMEM/F12) supplemented with 10% fetal bovine serum (FBS), 1% l-glutamine, 50 U/mL penicillin, 50 mg/mL streptomycin, 50 μg/mL ascorbic acid, and 50 μM α-tocopherol (Euroclone, Milan, Italy). Cells were tested for contamination, including *Mycoplasma*, and used within 2–4 months. All experiments were performed with an 80% confluent monolayer. The protective effects of Hyt were studied with the acute toxicity model by pre-treating cells with Hyt@tgel for 24 and 96 h followed by 24 h H_2_O_2_ (230 μM) [38] treatment in the absence of hydroxytyrosol. A shorter exposure (4 h) was used to investigate effects on mRNA expression.

#### 2.5.2. Intracellular Oxidative Stress

DCFH-DA assay was used to measure the production of intracellular reactive oxygen species (ROS) in C20A4 cells according to the manufacturer’s protocol. Following treatment, cells were labeled with DCFH-DA (25 μM) for 1 h in the dark. The fluorescence was measured every 5 min for 1 h, with an excitation wavelength of 485 nm and an emission wavelength of 535 nm using a microplate reader (Cytation 3). 

The malondialdehyde (MDA) concentration, as a lipid peroxidation index, was determined using the thiobarbituric acid reactive substances (TBARS) assay, according to the manufacturer’s protocol. The basal concentration of MDA was established adding about 600 µL of TBARS solution to 50 µg of total protein dissolved in 300 µL of Milli-Q water. The mix was incubated for 40 min at 100 °C prior to centrifugation at 14,000 rpm for 2 min. The supernatant was analyzed with a microplate reader at a wavelength of 532 nm [39].

Total SOD-like activity was assessed with the SOD Assay Kit-WST according to the manufacturer’s protocol. The activity was expressed as units per mg of protein, where one unit of enzyme inhibits reduction of cytochrome C by 50% in a coupled system formed by xanthine and xanthine oxidase.

#### 2.5.3. Enzyme-Linked Immunosorbent Assay (ELISA)

Secreted IL-6, IL-8, and TNF-α protein levels were measured in supernatants of chondrocytes treated as reported in paragraph 2.5.1. Briefly, 100 μL of samples and standards were added into the wells already pre-coated with antibody specific for IL-6, IL-8, or TNF-α, and incubated for 2 h at 37 °C. Unbound substances were removed and 100 μL of biotin-conjugated antibody specific for IL-6, IL-8, or TNF-α was added to the well. After washing, 100 μL of avidin conjugated Horseradish Peroxidase (HRP) was added to the wells and incubated for 1 h at 37 °C, followed by addition of 90 μL of TMB substrate solution, and then incubation for 15–30 min at 37 °C. Stop solution was added to each well, the plate was gently tapped for thorough mixing, and the color intensity measured at 450 nm using a Cytation 3 Microplate Reader (ASHI, Milan, Italy).

#### 2.5.4. Quantitative Senescence-Associated Beta-Galactosidase Assay

4-methylumbelliferyl-β-D-galactopyranoside (4-MUG) was used as substrate of β-galactosidase for the quantitative SA-β-gal assay [40]. 4-MUG does not fluoresce until cleaved by the enzyme to generate the fluorophore 4-methylumbelliferone. The assay was carried out on lysates obtained from cells that were grown as reported above. The production of the fluorophore was monitored at an emission/excitation wavelength of 365/460 nm.

#### 2.5.5. RNA Isolation, Reverse Transcription, and Quantitative Real-Time PCR (qRT-PCR)

Total RNA was extracted from cell cultures using TriFast (EuroClone, Milan, Italy), according to the manufacturer’s protocol, and mRNA levels quantified by RT-PCR amplification as reported by Calarco el al. [41]. For retro-transcription, total RNA (0.5 μg) was treated as described in EuroClone standard protocol and amplified by qPCR. Specific primers for SRY-Box Transcription Factor 9 (*SOX9*), Collagen Type II Alpha 1 Chain (*COL2A1*), Aggrecan (*ACAN*), Cartilage Oligomeric Matrix Protein *(COMP*), Interleukin-6 (*IL-6*), Interleukin-8 (*IL-8*), tumor necrosis factor (*TNF*)-*α*, Matrix Metallopeptidase 3 and 13 (*MMP-3* and *13*), and β-Actin (*ACTB*) were used and listed in Table 1. qRT-PCR was run on a 7900 HT fast real-time PCR System (Applied Biosystem, Milan, Italy). The reactions were performed according to the manufacturer’s instructions using SYBR Green PCR Master mix (Euroclone, Italy). Data were normalized using the housekeeping gene (ACTB). All reactions were run in triplicate and the results expressed as mean ± SD. The 2^−ΔΔCt^ method was used to determine the relative quantification.

### 2.6. Statistical Analysis

Statistical comparisons between the different experimental groups and controls were made using GraphPad Prism 6 software (GraphPad Software Inc., San Diego, CA, USA). Each experiment was performed at least three times and all quantitative data are expressed as mean ± standard deviation (SD).

## 3. Results and Discussion

### 3.1. Preparation and Physicochemical Characterization of Hyt-Loaded Nanoparticles (Hyt NPs)

OA, the most common musculoskeletal disease in the elderly population, involves the inflammatory immune response at both local (joint site) and systemic levels leading to severe articular joint pain and reduced joint mobility. To date, local anti-inflammatory treatment is usually insufficient because of their short intra-articular half-lives, while systemic administration is associated with more adverse events [42,43,44]. Chitosan-based nanoparticles have been extensively used as ideal drug carriers for wide range of biomedical applications due to their good compatibility and degradability [45,46]. 

In this work, Hyt-loaded nanoparticles (Hyt NPs) were successfully produced by the ionic gelation method, using tripolyphosphate (TPP) as the crosslink. Although the ionic gelation process represents a simple and robust route to obtain chitosan NPs in aqueous medium and under mild conditions, the optimal process parameters were determined to achieve NPs with high drug loading and narrow polydispersity index (PDI). Indeed, the ratio of chitosan/TPP, the chitosan concentration, and the concentration of the encapsulated drug could interfere with the NP size and size distribution during NP formation. 

Results of polymer ratio on particle size and size distribution, polydispersity index (PDI), zeta potential (ZP), and encapsulation efficiency (EE%) of nine batches of Hyt NPs studied are summarized in Table 2. Particle size of the prepared formulations was in a nanometric range varying between 510.14 ± 13.21 nm (CS:TPP 1:1) and 137.56 ± 3.13 nm (CS:TPP 10:1) demonstrating that the size of the nanoparticles depends greatly on the ratio of CS to TPP. This behavior is achieved by the interaction of the phosphate charged groups of TPP with the –NH_3_^+^ groups within the CS structure. Indeed, as the amount of TPP increases the particle size decreases because of the increment in the cross-linking of CS macromolecules mediated by TPP, leading to a minimum particle size at 10:1 CS:TPP ratio. 

Moreover, all formulations present a narrow size distribution and high positive surface charge indicating their better stability to aggregation due to the repulsive forces exerted by the positive surface charge. As reported in Table 2, the EE of Hyt NPs enhanced with an increase in CS:TPP ratio ranging between 18.31 ± 1.23% of 1:1 and 74.18 ± 3.16% of 10:1. This could likely be attributed to the number of crosslinking units associated with different TPP concentrations [47]. Moreover, the reduction in nanoparticle size obtained with 10:1 CS:TPP ratio resulted in increment of space for drug encapsulation.

According to the above results, the chitosan and TPP ratio of 10:1 was chosen for further study as the obtained Hyt NPs showed the highest EE with acceptable particle size and distribution.

**Figure 1:**
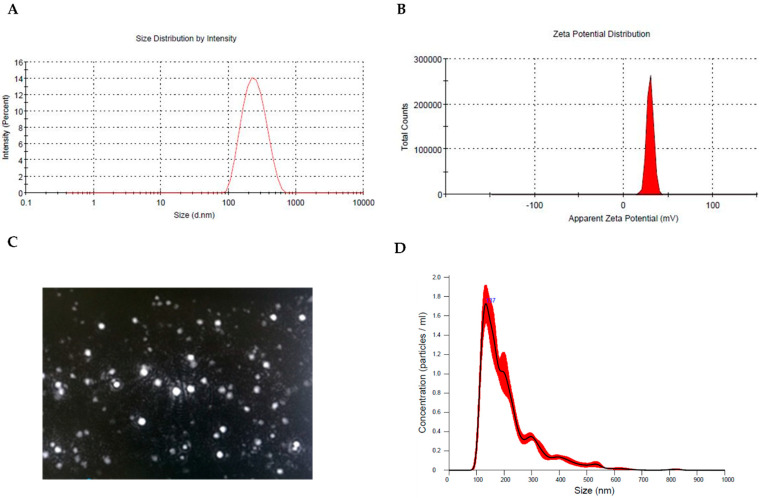
Physicochemical properties of Hyt-NPs. (**A**) Size distribution, (**B**) zeta potential profile, (**C**) screenshot of representative NTA video, and (**D**) NTA measurements for Hyt-NPs in suspension. Histograms are the average of 3 measurements.

Figure 1 shows representative images of: size (1A), zeta potential (1B) distribution, screenshot of nanoparticles tracking analysis video (NTA, 1C), and measurements (1D) of Hyt-NPs synthetized in the optimal condition.

### 3.2. In Vitro Hydrogel Formulation (Hyt@tgel) and Hyt Release

**Figure 2:**
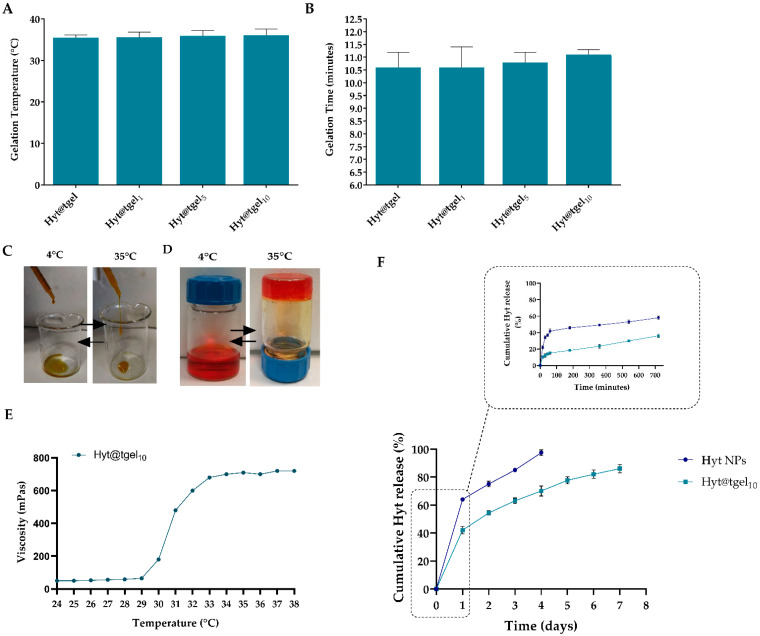
Characterizations of Hyt@tgel. Gelation temperature (**A**) and gelation time at 37 °C (**B**) of different hydrogel compositions (Hyt@tgel, Hyt@tgel_1_, Hyt@tgel_5_, and Hyt@tgel_10_). Representative photographs of the Hyt@tgel_10_ syringeability (**C**) and inverted test tube (**D**) obtained at 4 and 35 °C. Phenol red was added to facilitate hydrogel monitoring. (**E**) Solution viscosity measurement of the Hyt@tgel_10_ as a function of temperature. (**F**) Cumulative Hyt release from Hyt NPs and Hyt@tgel_10_ in phosphate buffer saline (PBS) after 24 h (and eight days). Six different experiments were conducted, and the results expressed as the mean of the values obtained (mean ± SD).

To obtain a sustained and localized drug delivery of Hyt at body temperature, different amounts of Hyt NPs were dispersed into injectable hydrogels composed of 20 wt% of Pluronic F127 and 1 wt% Hyaluronic acid (Hyt@tgel). According to Young-seok et al. [33] the Hyt@tgel formulation was optimized to reduce the Pluronic F-127 concentration needed to obtain gelation at body temperature. Moreover, the hydrophobic interaction between acetyl groups on HA and methyl groups on Pluronic could enhance the mechanical strength of the resulting hydrogel at temperatures above the critical gelation temperature (CGT). As shown in Figure 2A, the addition of different concentrations of Hyt-NPs (1, 5, and 10 mg) did not significantly affect the Hyt@tgel gelation temperature, suggesting that hydrogel structure organization was maintained after nanoparticles dispersion. These results are in agreement with previous studies at the same Pluronic concentrations [48,49]. The gelation time of the Hyt@tgel at 35 °C was slightly increased by Hyt NPs incorporation (Figure 2B). In particular, the presence of high nanoparticle concentrations increases the gelation time by 0.5 min (10 mg, Hyt@tgel_10_) and 0.2 min (5 mg, Hyt@tgel_5_) with respect to Hyt@tgel alone (10.6 min). Long in vivo gelation time, in fact, can cause nanoparticle loss by diffusion into the surrounding tissue. On the contrary, gelation that occurs too quickly could lead to clogging of the injection needle resulting in incomplete administration. Based on suitable gelation time and temperature, further analyses were conducted only on the Hyt@tgel_10_ sample. As shown in Figure 2C, Hyt@tgel_10_ demonstrated easy injectability through hypodermic needles at room temperature, while when the temperature increases at 35 °C, the extrusion needs the application of an extra force due to the increase in the viscosity. When the gel concentration reached the critical gelation concentration, the Hyt@tgel_10_ passed from an aqueous solution to a gel as the temperature was increased from 4 to 35 °C as demonstrated by the inversion test tube (Figure 2D). Hyt@tgel_10_ exhibited a viscous flowable form at low temperature becoming a semi-solid gel after incubation at temperature higher than 30 °C. This behavior was confirmed by the measure of viscosity as a function of temperatures (Figure 2E). 

There is a substantial body of evidence that encapsulation enhances the bioactivity of compounds improving their stability in aqueous medium and increasing upon the delivery at the target site. Chen et al. demonstrated the ability of chitosan microspheres dispersed in a thermally responsive chitosan hydrogel to load anti-inflammatory drugs. After injection into the knee joints of OA rabbits, drugs were released for more than 7 days in a controlled manner [50]. According to Wang et al., curcumin-loaded HA/chitosan nanoparticles exhibited a good sustained-release property leading to inflammation and cartilage apoptosis inhibition acting on the NF-κB pathway [51]. 

Chitosan nanoparticles have been recognized as a useful drug delivery tool in OA for their ability to prolong the drug retention time. To evaluate the sustained release properties of Hyt-NPs, an in vitro drug release study of Hyt from Hyt-NPs and from HYt@tgel_10_ was carried out using dialysis membrane against phosphate buffer saline (PBS). As shown in Figure 2F, the in vitro release of Hyt by chitosan NPs exhibited a fast drug release rate with 41% of Hyt released within the first hour, with the majority of the release occurring during the initial 2 days (75%). On the contrary, Hyt release rate from Hyt@tgel_10_ significantly slowed down (*p* < 0.05) with only 10% of Hyt released after 1 h, followed by a prolonged Hyt release up to 1 week. The slow Hyt release from the hydrogel could be attributed to the densely packed inter-micellar structure due to the presence of HA. Moreover, the highly packed super-molecular structure could reduce the diffusion coefficients inside of the hydrogel leading to a prolonged drug release.

Physical stability of nanoparticles in Hyt@tgel was investigated at 4 and 25 °C over 14 days by measuring size and PDI. As reported in Table 3, Hyt NPs were stable when stored at both low and room temperatures without a significant increase in particle size and PDI. Moreover, the drug encapsulation efficiency, assessed in parallel, demonstrated no decrease in the Hyt retention rate over the 14-day period confirming the protective effect of chitosan nanoparticles on biomolecules.

Taken together, the physicochemical behavior of Hyt@tgel_10_ is consistent with a potential use as a device to be injected through a syringe, because the sol-to-gel transition temperature is between room temperature and physiological temperature.

### 3.3. Oxidative Damage Protection of Hyt@tgel 

**Figure 3:**
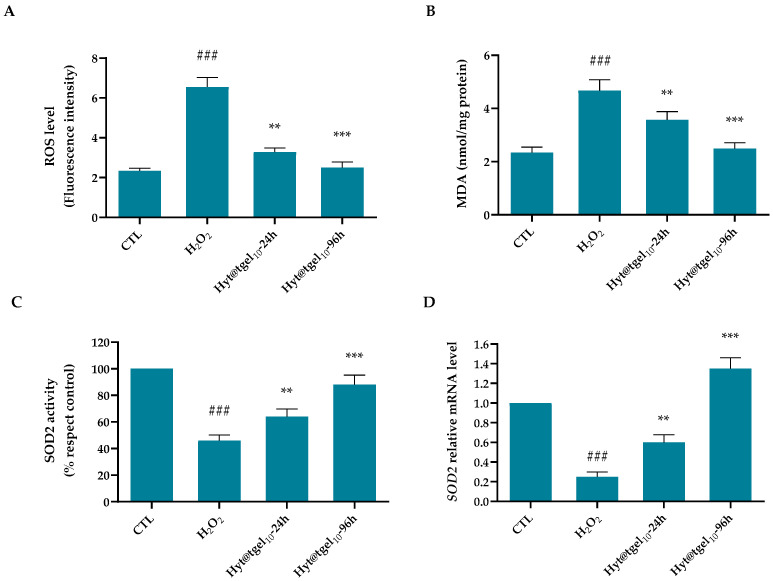
Antioxidant capacity of Hyt@tgel in H_2_O_2_-treated chondrocytes. C20A4 cells were incubated in the presence of hydrogel for 24 and 96 h and then treated with hydrogen peroxide for 24 h. (**A**) ROS release was determined by oxidized H2DCFDA (DCF). (**B**) Malondialdehyde quantity was used as a marker of lipid peroxidation. (**C**) Superoxide dismutase (SOD2) activity measured by assay kit. (**D**) SOD2 mRNA transcription level. Results are expressed as the mean of three independent experiments ± S.D (*n* = 3). ** *p* < 0.01, *** *p* < 0.001 versus untreated cells (control). ### *p* < 0.001 versus H_2_O_2_ group.

Several studies have concluded that OA progression is significantly related to an imbalance between the production of reactive oxygen species (ROS) and their clearance by an antioxidant defense system [52,53]. During OA pathogenesis, chondrocytes become both source and target of elevated amounts of reactive chemical species, particularly oxygen and nitrogen species triggering a vicious circle that leads to further damage of cartilage cells and matrix [54]. A wide body of evidence suggested that Hyt has antioxidant activity by inhibition and/or scavenging of reactive oxygen species (ROS) [54,55]. Moreover, a gene expression profiling study has suggested that Hyt affect the expression of genes involved in oxidative stress, inflammation, cell proliferation, or differentiation, suggesting that the beneficial effects of this molecule may be multifactorial and context-dependent [56]. The efficiency of Hyt@tgel_10_ to reduce the intracellular ROS generation was assessed in C20A4 cells in the presence of hydrogen peroxide (H_2_O_2_) (Figure 3A,B). The stimulation of chondrocytes with H_2_O_2_ mimics the in vivo condition observed in OA cartilage inducing the production of cellular and mitochondrial ROS and producing proinflammatory and procatabolic responses [57,58]. A significant increase (*p* < 0.001) in chondrocyte intracellular oxidants by approximately 2.8 times was obtained with respect to untreated cells (Control) after H_2_O_2_ 24 h treatment (Figure 3A). A short-time pre-incubation (24 h) with Hyt@tgel_10_ considerably reduced (*p* < 0.01) H_2_O_2_-induced ROS production of about 1.4-fold with respect to the H_2_O_2_ group. Moreover, the protective effect of released Hyt was greatly enhanced ((*p* < 0.001) by a longer pre-treatment (96 h) resulting in slight fluorescence increase with respect to control cells. MDA, a lipid peroxidation end product, is abundant in synoviocytes from patients with OA. Under oxidative stress, polyunsaturated fatty acids of cellular membrane lipids represent the prime targets of ROS attack. The lipid peroxidation leads to the formation of chemically reactive lipid aldehydes, such as MDA, capable of causing severe damage to nucleic acids and proteins, altering their functions and leading to the loss of both structural and metabolic function of cells [59]. As reported in Figure 3B, treatment of cells with H_2_O_2_ increased intracellular lipid peroxidation to 2-fold relative to control (*p* < 0.001). Conversely, the presence of Hyt@tgel_10_ for 24 h markedly diminished (*p* < 0.01) the MDA level (1.1-fold) compared with H_2_O_2_ treated cells, with a marked decrease (*p* < 0.001) after 96 h leading the MDA formation to levels almost similar to control. 

To prevent an accumulation of ROS-mediated damage, chondrocytes produce a number of antioxidant enzymes including the superoxide dismutases (SOD), catalase, and glutathione peroxidase [60]. The three SOD family members SOD1, SOD2, and SOD3 transform O_2_^−^ into hydrogen peroxide (H_2_O_2_), limiting the formation of highly aggressive compounds such as ONOO^−^ and OH^−^. All SOD are expressed at lower levels in OA cartilage compared to normal control cartilage, at both the messenger RNA (mRNA) and protein level. In particular, Ruiz-Romero et al. demonstrated through a proteomics approach, a significant decrease in the major mitochondrial antioxidant protein manganese-superoxide dismutase (SOD2) in the superficial layer of OA cartilage. This SOD2 reduction makes cartilage more susceptible to ROS damage suggesting a central role of mitochondrial redox imbalance in OA pathogenesis [61]. To verify if the antioxidant actions of Hyt have been related not only to its free radical scavenging activity, but also to the ability to enhance the endogenous defense system by inducing antioxidant/detoxifying enzymes activity, SOD2 activity was assayed. As shown in Figure 3C, treatment with H_2_O_2_ leads to decrease in antioxidant enzyme activity of about 54% with respect to untreated cells. When chondrocytes were pre-treated with Hyt@tgel_10_ for 24 and 96 h, SOD2 activity was 18% and 42%, respectively, higher than that in H_2_O_2_-treated cells, demonstrating a good ability to protect mitochondria from oxidative damage. Moreover, Hyt@tgel_10_ pretreatment restored the SOD2 transcript to above their control levels, by significantly increasing its expression by 2.4-fold for 24 h and 5.5-fold after 96 h over the H_2_O_2_-depressed level (Figure 3D). 

Taken together, the results reported herein confirm a key role of Hyt@tgel_10_ pre-treatment to effectively suppress the production of intracellular ROS and lipid peroxidation and also elevated the activity of antioxidant enzymes such as SOD, limiting oxidative stress-induced damage in the OA in vitro model. 

### 3.4. Hyt@tgel Suppresses Inflammatory Response in Chondrocytes

**Figure 4:**
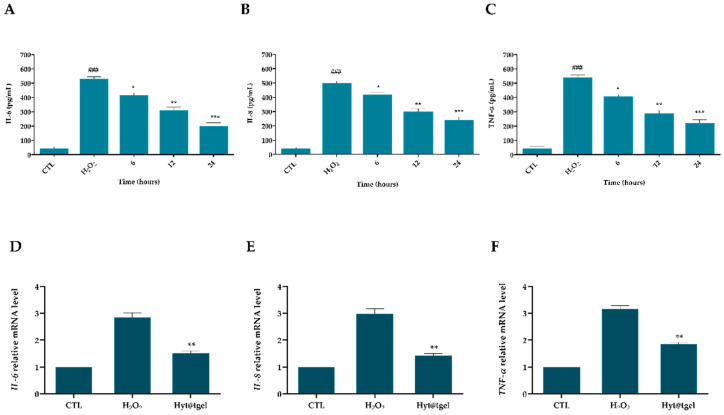
Hyt@tgel_10_ inhibits H_2_O_2_-induced inflammatory response in chondrocytes. The effect of Hyt on the production of IL-6 (**A**,**D**), IL-8 (**B**,**E**), and TNF-α (**C**,**F**) was measured by ELISA assay (**A**–**C**) and qRT-PCR (**D–F**). C20A4 cells were pre-treated with Hyt@tgel_10_ for 24 h, then stimulated with H_2_O_2_ for 24 h (ELISA assay) or 4 h (qRT-PCR). Results are expressed as the mean of three independent experiments ± S.D (*n* = 3). ### *p* < 0.001 H_2_O_2_ vs. CTL, * *p* < 0.05, ** *p* < 0.01, and *** *p* < 0.005 Hyt@tgel_10_ vs. H_2_O_2_.

Increases in the levels of the cytokines in joints plays a central role in the pathogenesis of OA by modulating oxidative stress, cartilage ECM turnover, and chondrocytes apoptosis [62,63]. The current drugs for treating OA are developed primarily to relieve pain and control symptoms, failing to cure the disease [63]. Epidemiologic studies demonstrated the lower incidence of inflammatory chronic disease, such as OA in people of the Mediterranean basin. One of the possible reasons is that Mediterranean people have a high intake of olive and olive oil rich in polyphenolic compounds with antioxidant and anti-inflammatory properties [14,64,65]. During the pathophysiological processes of OA, cytokines, hormone-like proteins, are responsible for the loss of metabolic homeostasis of tissues forming joints by promoting catabolic and destructive processes. Olive-oil-rich extracts inhibit the production of proinflammatory cytokines, including IL-1β, TNF-α, IL-6, and prostaglandin E2 in arthritic joints [66,67]. Richard and colleagues demonstrated a pivotal role of Hyt extracted from olive vegetation water in diminished secretion of cytokines (IL-1 α, IL-1 β, IL-6, IL-12, TNF-α), and chemokines (CXCL10/IP-10, CCL2/MCP-1) in murine macrophages (RAW264.7 cells) stimulated with lipopolysaccharide (LPS) [68]. Another study showed a decrease in the severity of the disease and an overall anti-IL-1β effect after treatment with olive and grape seed extract in animal models of post-traumatic OA [69]. In the present study, secreted IL-6, IL-8, and TNF-α were detected in the supernatant of chondrocytes cell line C20A4 by enzyme-linked immunosorbent assays (ELISA). As expected, incubation of cells for 24 h with Hyt@tgel_10_ significantly reduces the amount of released cytokines with respect to the control in a time-dependent manner (Figure 4A–C). Consistently, the protective effects of Hyt were confirmed also by RT-qPCR analysis. As reported in Figure 4D–F, the mRNA levels of all tested cytokines (relative to the housekeeping gene) were significantly upregulated (*p* < 0.01) in H_2_O_2_-treated cells, compared with the control group. As expected, the H_2_O_2_-driven release of IL-6, IL-8, and TNF-α was decreased by Hyt@tgel_10_ pre-treatment with a 50% reduction in interleukin expression levels with respect to H_2_O_2_-treated cells. 

### 3.5. Hyt@tgel Protects against H_2_O_2_-Mediated Chondrocyte ECM Degradation

**Figure 5:**
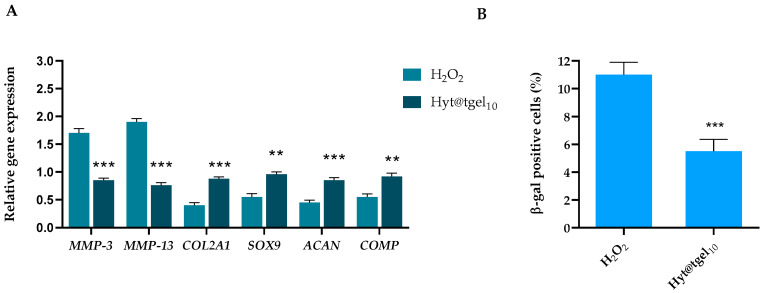
Hyt released by Hyt@tgel_10_ prevents the expression of OA-related genes in chondrocytes treated with H_2_O_2_. (**A**) C20A4 chondrocytes were pre-treated with Hyt@tgel_10_ for 24 h, then stimulated with H_2_O_2_ for 4 h (±SD, *n* = 3, ** *p* < 0.01 vs. H_2_O_2_ group). (**B**) Beta-galactosidase senescence assay. The graph shows the mean percentage value of senescent cells in every experimental condition (±SD, *n* = 3, *** *p* < 0.005).

Once damaged, the cartilage is enabled to repair itself due to its special physiological structure. In the early stages of OA, the production of inflammatory mediators including cytokines and prostaglandins by the cartilage and synovial cells lead to activation of matrix metalloproteinases (MMPs) [70]. Among them, matrix metalloproteinases MMP-3 and MMP-13 can further promote cartilage inflammation, chondrocyte apoptosis, and ROS production, via a positive feedback loop [71,72]. Emerging evidence has shown that MMP13 is considered a significant biomarker to assess OA therapeutic effects and OA progression [73,74]. In this context, bioactive molecules able to suppress these inflammatory mediators or block the involved signaling pathway may help to reduce the OA pathological process [75]. To further corroborate the Hyt@tgel anti-inflammatory action, the expression of catabolic genes such as those coding for MMP-3 and MMP-13 were evaluated (Figure 5). 

Compared with the untreated group, the mRNA expression of MMP-3 and MMP-13 increased significantly (*p* < 0.01) in H_2_O_2_-treated cells, while Hyt@tgel_10_ pre-treatment was able to reduce about 40% of the increases provoked by H_2_O_2_. These results, in line with Facchini et al. [25] corroborated the capacity of Hyt to antagonize the activation of pro-inflammatory pathways like NF-κB even in chondrocytes. 

Activation of catabolic enzymes degrades proteoglycan and collagen in the articular cartilage. Moreover, inflammatory states lead to de-differentiation of chondrocytes accompanied by decreased expression of chondrocyte-specific proteins [76]. As reported in Figure 5A, the expression of SOX9 [77] (an early marker of the formation of a cartilage-like tissue matrix), COL2A1, ACAN [78], and COMP (markers of the final stage of chondrogenic differentiation) was significantly rescued by incubation with Hyt@tgel_10_. These data indicate that Hyt released by Hyt@tgel influenced the ECM balance and gene expression in the chondrocytes under pathological state maintaining their metabolic activity and proliferation in their differentiated phenotype. 

Although various cell types are involved in OA pathology, chondrocytes play a major role in OA induction by cellular senescence [79]. It has been shown that chondrocytes have telomere shortening with age. For this reason, chondrocyte senescence, caused by chronic stress in the cells or caused by post-traumatic inflammation, is believed to be closely related to OA [80]. Therefore, the regulation of hypertrophic or senescent chondrocytes using natural phytochemicals known to have a powerful anti-inflammatory and antioxidant activity could be a potential therapeutic target to slow or stop the progression of OA [76]. Data demonstrated that senescence was noticeably reduced in cells treated with Hyt@tgel as detected in an in-situ beta-galactosidase assay (Figure 5B). In particular, Hyt@tgel_10_ treatment reduced more than two times the percentage of senescent cells compared to untreated chondrocytes. 

Two different mechanisms of senescence are suggested in chondrocytes: replicative senescence and stress-induced premature senescence [81,82]. Upregulation of inflammatory cytokines expression induces senescence directly, while downregulation of chondrocyte phenotypic maintenance genes such as SOX9, BMP-2, IGF-1, and ACAN induces senescence indirectly. Thus, the association between aging and/or trauma, reduces the number of healthy and functioning chondrocytes, promoting cartilage degeneration and eventually leads to osteoarthritic pathophysiology [10]. Therefore, the reduction of this cell population lends further credit to hydroxytyrosol ability to preserve chondrocytes from senescence after Hyt@tgel_10_ treatment. 

## 4. Conclusions

OA is mainly caused by trauma induced by an external force or cartilage damage ac-cumulated during aging. This study provided new insights into the therapeutic effects of intra-articular injection of Hyt-loaded chitosan nanoparticles embedded into thermosensitive hydrogels (Hyt@tgel_10_). The hydrogel exhibited a sol-gel transition behavior and a gelation time consistent with its therapeutic application. Moreover, Hyt released from hydrogel was able to protect chondrocytes from ROS damage and to revert the activation of inflammatory factors, limiting, in an in vitro model, the vicious cycle typical of OA progression. Hence, it can be concluded that the formulated hydrogel injection could be proposed for the efficient and sustained Hyt delivery for OA treatment. The next step would be the extraction of “added-value” bioactive polyphenols from by-products of the olive industry, in order to develop a green delivery system able not only to enhance human wellbeing but also to promote a sustainable environment.

## Figures and Tables

**Table 1:** Primers used for qRT-PCR.

Gene	Accession Number	Forward (5′-3′)	Reverse (5′-3′)
*COL2A1*	NM_001844.5	CTGGTGTGAAGGGTGAGAGT	AGTCCGTCCTCTTTCACCAG
*ACAN*	NM_001135.4	TCCCCAACAGATGCTTCCAT	GTACTTGTTCCAGCCCTCCT
*SOX9*	NM_000346.4	CCGCTCACAGTACGACTACA	GTGAAGGTGGAGTAGAGGCC
*COMP*	NM_000095.4	CCTTCAATGGCGTGGACTTC	TGACCACGTAGAAGCTGGAG
*MMP-3*	NM_002422.5	CCTCTGATGGCCCAGAATTGA	GAAATTGGCCACTCCCTGGGT
*MMP-13*	NM_002427.4	GTCCAGGAGATGAAGACCCC	CTCGGAGACTGGTAATGGCA
*SOD2*	NM_000636.4	CTGGACAAACCTCAGCCCTA	TGATGGCTTCCAGCAACTC
*IL-6*	NM_000600.5	CGCCTTCGGTCCAGTTGCC	GCCAGTGCCTCTTTGCTGCTTT
*IL-8*	NM_000584.4	CTCTTGGCAGCCTTCCTGATTTC	TTTTCCTTGGGGTCCAGACAGAG
*TNF-α*	NM_000594.4	AACATCCAACCTTCCCAAACGC	TGGTCTCCAGATTCCAGATGTCAGG
*ACTB*	NM_001101.5	ACTCTTCCAGCCTTCCTTCC	CGTACAGGTCTTTGCGGATG

**Table 2:** Effect of chitosan concentration and chitosan/TPP ratio on the size (hydrodynamic diameter), polydispersity index (PDI), zeta potential (ZP), and encapsulation efficiency (EE) of Hyt-loading nanoparticles (Hyt NPs). The Hyt concentration was kept constant at 10 mg.

Chitosan (mg/mL)	CS:TPP Mass Ratio	Size (nm ± SD)	PDI (mV ± SD)	EE (% ± SD)
0.1	1:1	510.14 ± 13.21	0.28 ± 0.03	18.31 ± 1.23
0.1	5:1	365.23 ± 9.01	0.15 ± 0.04	36.71 ± 1.26
0.1	10:1	298.73 ± 9.06	0.26 ± 0.02	39.27 ± 2.14
0.5	1:1	330.33 ± 7.52	0.17 ± 0.01	28.96 ± 1.34
0.5	5:1	279.61 ± 0.72	0.23 ± 0.04	41.25 ± 2.41
0.5	10:1	219.63 ± 0.46	0.21 ± 0.03	53.37 ± 2.62
1	1:1	348.16 ±12.64	0.14 ± 0.02	29.81 ± 1.29
1	5:1	224.43 ± 0.46	0.25 ± 0.01	48.57 ± 1.85
1	10:1	137.56 ± 3.13	0.16 ± 0.03	74.18 ± 3.16

Note: In the same column, value with the same subscript letter (a–c) were not significantly different (*p* > 0.05). Data were mean of three replications ± standard deviation (SD).

**Table 3:** Particle size and entrapment efficiency studies of Hyt@tgel_10_ before and after 14-day storage.

	Hyt@tgel_10_ Before Storage	Hyt@tgel_10_ after 14-Day Storage at 4 ± 1 °C	Hyt@tgel_10_ after 14-Day Storage at 25 ± 1 °C
Average particle size (nm)	137.56 ± 3.13	135.32 ± 2.56 nm	139.00 ± 6.53 nm
PDI	0.16 ± 0.03	0.15 ± 0.02	0.18 ± 0.05
Entrapment efficiency (% EE)	74.18 ± 3.16	75.88 ± 4.13	77.22 ± 5.34

Note: Data were expressed as mean ± standard deviation, *n* = 3.

## Data Availability

The data presented in this study are available in the article.

## References

[1] Roos E., Arden N.K. (2016). Strategies for the prevention of knee osteoarthritis. Nat. Rev. Rheumatol..

[2] Zheng L., Zhang Z., Sheng P., Mobasheri A. (2021). The role of metabolism in chondrocyte dysfunction and the progression of osteoarthritis. Ageing Res. Rev..

[3] Li G.Y., Yin J.M., Gao J.J., Cheng T.S., Pavlos N.J., Zhang C.Q., Zheng M.H. (2013). Subchondral bone in osteoarthritis: Insight into risk factors and microstructural changes. Arthritis Res. Ther..

[4] Martel-Pelletier J., Barr A., Cicuttini F., Conaghan P., Cooper C., Goldring M.B., Goldring S.R., Jones G., Teichtahl A.J., Pelletier J.-P. (2016). Osteoarthritis. Nat. Rev. Dis. Prim..

[5] Ameye L.G., Chee W.S. (2006). Osteoarthritis and nutrition. From nutraceuticals to functional foods: A systematic review of the scientific evidence. Arthritis Res. Ther..

[6] Bijlsma J.W., Berenbaum F., Lafeber F.P. (2011). Osteoarthritis: An update with relevance for clinical practice. Lancet.

[7] Lepetsos P., Papavassiliou A.G. (2016). ROS/oxidative stress signaling in osteoarthritis. Biochim. Biophys. Acta (BBA)-Mol. Basis Dis..

[8] Zahan O.-M., Serban O., Gherman C., Fodor D. (2020). The evaluation of oxidative stress in osteoarthritis. Med. Pharm. Rep..

[9] Bolduc J.A., Collins J.A., Loeser R.F. (2019). Reactive oxygen species, aging and articular cartilage homeostasis. Free Radic. Biol. Med..

[10] Ramasamy T.S., Yee Y.M., Khan I.M. (2021). Chondrocyte Aging: The Molecular Determinants and Therapeutic Opportunities. Front. Cell Dev. Biol..

[11] Fernandes L., Hagen K.B., Bijlsma J.W.J., Andreassen O., Christensen P., Conaghan P., Doherty M., Geenen R., Hammond A., Kjeken I. (2013). EULAR recommendations for the non-pharmacological core management of hip and knee osteoarthritis. Ann. Rheum. Dis..

[12] Miles E.A., Zoubouli P., Calder P. (2005). Differential anti-inflammatory effects of phenolic compounds from extra virgin olive oil identified in human whole blood cultures. Nutrition.

[13] Finicelli M., Squillaro T., Di Cristo F., Di Salle A., Melone M.A.B., Galderisi U., Peluso G. (2019). Metabolic syndrome, Mediterranean diet, and polyphenols: Evidence and perspectives. J. Cell. Physiol..

[14] Finicelli M., Squillaro T., Galderisi U., Peluso G. (2021). Polyphenols, the Healthy Brand of Olive Oil: Insights and Perspectives. Nutrients.

[15] Valentino A., Di Cristo F., Bosetti M., Amaghnouje A., Bousta D., Conte R., Calarco A. (2021). Bioactivity and Delivery Strategies of Phytochemical Compounds in Bone Tissue Regeneration. Appl. Sci..

[16] Del Rio D., Rodriguez-Mateos A., Spencer J.P.E., Tognolini M., Borges G., Crozier A. (2013). Dietary (Poly)phenolics in Human Health: Structures, Bioavailability, and Evidence of Protective Effects Against Chronic Diseases. Antioxid. Redox Signal..

[17] De Luca I., Di Cristo F., Valentino A., Peluso G., Di Salle A., Calarco A. (2022). Food-Derived Bioactive Molecules from Mediterranean Diet: Nanotechnological Approaches and Waste Valorization as Strategies to Improve Human Wellness. Polymers.

[18] Visioli F., De La Lastra C.A., Andres-Lacueva C., Aviram M., Calhau C., Cassano A., D’Archivio M., Faria A., Favé G., Fogliano V. (2011). Polyphenols and Human Health: A Prospectus. Crit. Rev. Food Sci. Nutr..

[19] Silva A.F.R., Resende D., Monteiro M., Coimbra M.A., Silva A.M.S., Cardoso S.M. (2020). Application of Hydroxytyrosol in the Functional Foods Field: From Ingredient to Dietary Supplements. Antioxidants.

[20] Serra A., Rubió L., Borràs X., Macià A., Romero M.-P., Motilva M.-J. (2012). Distribution of olive oil phenolic compounds in rat tissues after administration of a phenolic extract from olive cake. Mol. Nutr. Food Res..

[21] Yonezawa Y., Miyashita T., Nejishima H., Takeda Y., Imai K., Ogawa H. (2018). Anti-inflammatory effects of olive-derived hydroxytyrosol on lipopolysaccharide-induced inflammation in RAW264.7 cells. J. Veter. Med. Sci..

[22] Di Meo F., Valentino A., Petillo O., Peluso G., Filosa S., Crispi S. (2020). Bioactive Polyphenols and Neuromodulation: Molecular Mechanisms in Neurodegeneration. Int. J. Mol. Sci..

[23] Fuccelli R., Fabiani R., Rosignoli P. (2018). Hydroxytyrosol Exerts Anti-Inflammatory and Anti-Oxidant Activities in a Mouse Model of Systemic Inflammation. Molecules.

[24] Cetrullo S., D’Adamo S., Guidotti S., Borzì R.M., Flamigni F. (2016). Hydroxytyrosol prevents chondrocyte death under oxidative stress by inducing autophagy through sirtuin 1-dependent and -independent mechanisms. Biochi. Biophys. Acta (BBA).

[25] Facchini A., Cetrullo S., D’Adamo S., Guidotti S., Minguzzi M., Facchini A., Borzì R.M., Flamigni F. (2014). Hydroxytyrosol Prevents Increase of Osteoarthritis Markers in Human Chondrocytes Treated with Hydrogen Peroxide or Growth-Related Oncogene α. PLoS ONE.

[26] Scalbert A., Williamson G. (2000). Dietary Intake and Bioavailability of Polyphenols. J. Nutr..

[27] Detsi A., Kavetsou E., Kostopoulou I., Pitterou I., Pontillo A.R.N., Tzani A., Christodoulou P., Siliachli A., Zoumpoulakis P. (2020). Nanosystems for the Encapsulation of Natural Products: The Case of Chitosan Biopolymer as a Matrix. Pharmaceutics.

[28] Le H., Xu W., Zhuang X., Chang F., Wang Y., Ding J. (2020). Mesenchymal stem cells for cartilage regeneration. J. Tissue Eng..

[29] Zha K., Sun Z., Yang Y., Chen M., Gao C., Fu L., Li H., Sui X., Guo Q., Liu S. (2021). Recent Developed Strategies for Enhancing Chondrogenic Differentiation of MSC: Impact on MSC-Based Therapy for Cartilage Regeneration. Stem Cells Int..

[30] Meng F.G., Zhang Z.Q., Huang G.X., Chen W.S., Zhang Z.J., He A.S., Liao W.M. (2016). Chondrogenesis of mesenchymal stem cells in a novel hyaluronate-collagen-tricalcium phosphate scaffolds for knee repair. Eur. Cells Mater..

[31] Arrich J., Piribauer F., Mad P., Schmid D., Klaushofer K., Müllner M. (2005). Intra-articular hyaluronic acid for the treatment of osteoarthritis of the knee: Systematic review and meta-analysis. Can. Med. Assoc. J..

[32] Reichenbach S., Blank S., Rutjes A.W.S., Shang A., King E.A., Dieppe P.A., Jüni P., Trelle S. (2007). Hylan versus hyaluronic acid for osteoarthritis of the knee: A systematic review and meta-analysis. Arthritis Rheum..

[33] Jung Y.-S., Park W., Park H., Lee D.-K., Na K. (2017). Thermo-sensitive injectable hydrogel based on the physical mixing of hyaluronic acid and Pluronic F-127 for sustained NSAID delivery. Carbohydr. Polym..

[34] Calvo P., Remuñán-López C., Vila-Jato J.L., Alonso M.J. (1997). Novel hydrophilic chitosan-polyethylene oxide nanoparticles as protein carriers. J. Appl. Polym. Sci..

[35] Conte R., Valentino A., Di Cristo F., Peluso G., Cerruti P., Di Salle A., Calarco A. (2020). Cationic Polymer Nanoparticles-Mediated Delivery of miR-124 Impairs Tumorigenicity of Prostate Cancer Cells. Int. J. Mol. Sci..

[36] Khattab A., Marzok S., Ibrahim M. (2019). Development of optimized mucoadhesive thermosensitive pluronic based in situ gel for controlled delivery of Latanoprost: Antiglaucoma efficacy and stability approaches. J. Drug Deliv. Sci. Technol..

[37] Amaghnouje A., Mechchate H., Es-Safi I., Boukhira S., Aliqahtani A.S., Noman O.M., Nasr F.A., Conte R., Calarco A., Bousta D. (2020). Subacute Assessment of the Toxicity and Antidepressant-Like Effects of *Origanum majorana* L. Polyphenols in Swiss Albino Mice. Molecules.

[38] Scuruchi M., D’Ascola A., Avenoso A., Mandraffino G., Campo S., Campo G.M. (2021). Endocan, a novel inflammatory marker, is upregulated in human chondrocytes stimulated with IL-1 beta. Mol. Cell. Biochem..

[39] Di Cristo F., Valentino A., De Luca I., Peluso G., Bonadies I., Calarco A., Di Salle A. (2022). PLA Nanofibers for Microenvironmental-Responsive Quercetin Release in Local Periodontal Treatment. Molecules.

[40] Musto P., Calarco A., Pannico M., La Manna P., Margarucci S., Tafuri A., Peluso G. (2017). Hyperspectral Raman imaging of human prostatic cells: An attempt to differentiate normal and malignant cell lines by univariate and multivariate data analysis. Spectrochim. Acta Part A Mol. Biomol. Spectrosc..

[41] Calarco A., Di Salle A., Tammaro L., De Luca I., Mucerino S., Petillo O., Riccitiello F., Vittoria V., Peluso G. (2015). Long-Term Fluoride Release from Dental Resins Affects STRO-1+ Cell Behavior. J. Dent. Res..

[42] Xia B., Chen D., Zhang J., Hu S., Jin H., Tong P. (2014). Osteoarthritis Pathogenesis: A Review of Molecular Mechanisms. Calcif. Tissue Res..

[43] Anandacoomarasamy A., March L. (2010). Current evidence for osteoarthritis treatments. Ther. Adv. Musculoskelet. Dis..

[44] Nowaczyk A., Szwedowski D., Dallo I., Nowaczyk J. (2022). Overview of First-Line and Second-Line Pharmacotherapies for Osteoarthritis with Special Focus on Intra-Articular Treatment. Int. J. Mol. Sci..

[45] Menazea A., Ahmed M. (2020). Wound healing activity of Chitosan/Polyvinyl Alcohol embedded by gold nanoparticles prepared by nanosecond laser ablation. J. Mol. Struct..

[46] Ali A., Ahmed S. (2018). A review on chitosan and its nanocomposites in drug delivery. Int. J. Biol. Macromol..

[47] Rathore P., Mahor A., Jain S., Haque A., Kesharwani P. (2020). Formulation development, in vitro and in vivo evaluation of chitosan engineered nanoparticles for ocular delivery of insulin. RSC Adv..

[48] Campos E.V.R., Proença P.L.F., da Costa T.G., de Lima R., Fraceto L.F., de Araujo D.R. (2022). Using Chitosan-Coated Polymeric Nanoparticles-Thermosensitive Hydrogels in association with Limonene as Skin Drug Delivery Strategy. BioMed Res. Int..

[49] Conte R., De Luise A., Valentino A., Di Cristo F., Petillo O., Riccitiello F., Di Salle A., Calarco A., Peluso G. (2018). Chapter 10—Hydrogel Nanocomposite Systems: Characterization and Application in Drug-Delivery Systems. Nanocarriers for Drug Delivery.

[50] Chen Z.-P., Liu W., Liu D., Xiao Y.-Y., Chen H.-X., Chen J., Li W., Cai H., Li W., Cai B.-C. (2012). Development of brucine-loaded microsphere/thermally responsive hydrogel combination system for intra-articular administration. J. Control. Release.

[51] Wang S., Wei X., Sun X., Chen C., Zhou J., Zhang G., Wu H., Guo B., Wei L. (2018). A novel therapeutic strategy for cartilage diseases based on lipid nanoparticle-RNAi delivery system. Int. J. Nanomed..

[52] Ansari M.Y., Ahmad N., Haqqi T.M. (2020). Oxidative stress and inflammation in osteoarthritis pathogenesis: Role of polyphenols. Biomed. Pharmacother..

[53] Amrati F.E.-Z., Bourhia M., Slighoua M., Ibnemoussa S., Bari A., Ullah R., Amaghnouje A., Di Cristo F., El Mzibri M., Calarco A. (2020). Phytochemical Study on Antioxidant and Antiproliferative Activities of Moroccan Caralluma europaea Extract and Its Bioactive Compound Classes. Evid. Based Complement. Altern. Med..

[54] Zhuo Q., Yang W., Chen J., Wang Y. (2012). Metabolic syndrome meets osteoarthritis. Nat. Rev. Rheumatol..

[55] Sarsour E.H., Kumar M.G., Kalen A.L., Goswami M., Buettner G.R., Goswami P.C. (2011). MnSOD activity regulates hydroxytyrosol-induced extension of chronological lifespan. AGE.

[56] Kim Y., Choi Y., Park T. (2010). Hepatoprotective effect of oleuropein in mice: Mechanisms uncovered by gene expression profiling. Biotechnol. J..

[57] Ansari M.Y., Khan N.M., Ahmad I., Haqqi T.M. (2018). Parkin clearance of dysfunctional mitochondria regulates ROS levels and increases survival of human chondrocytes. Osteoarthr. Cartil..

[58] Salgado C., Jordan O., Allémann E. (2021). Osteoarthritis In Vitro Models: Applications and Implications in Development of Intra-Articular Drug Delivery Systems. Pharmaceutics.

[59] Shi Q., Vaillancourt F., Côté V., Fahmi H., Lavigne P., Afif H., Di Battista J.A., Fernandes J.C., Benderdour M. (2006). Alterations of metabolic activity in human osteoarthritic osteoblasts by lipid peroxidation end product 4-hydroxynonenal. Arthritis Res. Ther..

[60] Henrotin Y., Kurz B., Aigner T. (2005). Oxygen and reactive oxygen species in cartilage degradation: Friends or foes?. Osteoarthr. Cartil..

[61] Ruiz-Romero C., Calamia V., Mateos J., Carreira V., Martínez-Gomariz M., Fernández M., Blanco F.J. (2009). Mitochondrial dysregulation of osteoarthritic human articular chondrocytes analyzed by proteomics: A decrease in mitochondrial superoxide dismutase points to a redox imbalance. Mol. Cell. Proteom..

[62] Kapoor M., Martel-Pelletier J., Lajeunesse D., Pelletier J.-P., Fahmi H. (2011). Role of proinflammatory cytokines in the pathophysiology of osteoarthritis. Nat. Rev. Rheumatol..

[63] Veronesi F., Della Bella E., Cepollaro S., Brogini S., Martini L., Fini M. (2016). Novel therapeutic targets in osteoarthritis: Narrative review on knock-out genes involved in disease development in mouse animal models. Cytotherapy.

[64] Ravalli S.M., Szychlinska M.A., Leonardi R.M., Musumeci G. (2018). Recently highlighted nutraceuticals for preventive management of osteoarthritis. World J. Orthop..

[65] Castrogiovanni P., Trovato F.M., Loreto C., Nsir H., Szychlinska M.A., Musumeci G. (2016). Nutraceutical Supplements in the Management and Prevention of Osteoarthritis. Int. J. Mol. Sci..

[66] Rosillo M., De La Lastra C.A., Castejón M.L., Montoya T., Cejudo-Guillén M., Sánchez-Hidalgo M. (2019). Polyphenolic extract from extra virgin olive oil inhibits the inflammatory response in IL-1β-activated synovial fibroblasts. Br. J. Nutr..

[67] Chin K.-Y., Pang K.-L. (2017). Therapeutic Effects of Olive and Its Derivatives on Osteoarthritis: From Bench to Bedside. Nutrients.

[68] Richard N., Arnold S., Hoeller U., Kilpert C., Wertz K., Schwager J. (2011). Hydroxytyrosol Is the Major Anti-Inflammatory Compound in Aqueous Olive Extracts and Impairs Cytokine and Chemokine Production in Macrophages. Planta Med..

[69] Mével E., Merceron C., Vinatier C., Krisa S., Richard T., Masson M., Lesoeur J., Hivernaud V., Gauthier O., Abadie J. (2016). Olive and grape seed extract prevents post-traumatic osteoarthritis damages and exhibits in vitro anti IL-1β activities before and after oral consumption. Sci. Rep..

[70] Charlier E., Deroyer C., Ciregia F., Malaise O., Neuville S., Plener Z., Malaise M., de Seny D. (2019). Chondrocyte dedifferentiation and osteoarthritis (OA). Biochem. Pharmacol..

[71] Musumeci G., Castrogiovanni P., Trovato F.M., Weinberg A.M., Al-Wasiyah M.K., Alqahtani M.H., Mobasheri A. (2015). Biomarkers of Chondrocyte Apoptosis and Autophagy in Osteoarthritis. Int. J. Mol. Sci..

[72] Musumeci G., Aiello F.C., Szychlinska M.A., Di Rosa M., Castrogiovanni P., Mobasheri A. (2015). Osteoarthritis in the XXIst Century: Risk Factors and Behaviours that Influence Disease Onset and Progression. Int. J. Mol. Sci..

[73] Wang M., Sampson E.R., Jin H., Li J., Ke Q.H., Im H.-J., Chen D. (2013). MMP13 is a critical target gene during the progression of osteoarthritis. Arthritis Res. Ther..

[74] Wan J., Zhang G., Li X., Qiu X., Ouyang J., Dai J., Min S. (2021). Matrix Metalloproteinase 3: A Promoting and Destabilizing Factor in the Pathogenesis of Disease and Cell Differentiation. Front. Physiol..

[75] Ryu J.-H., Yang S., Shin Y., Rhee J., Chun C.-H., Chun J.-S. (2011). Interleukin-6 plays an essential role in hypoxia-inducible factor 2α-induced experimental osteoarthritic cartilage destruction in mice. Arthritis Rheum..

[76] Benya P., Padilla S.R., Nimni M.E. (1978). Independent regulation of collagen types by chondrocytes during the loss of differentiated function in culture. Cell.

[77] Kawaguchi J., Mee P., Smith A. (2005). Osteogenic and chondrogenic differentiation of embryonic stem cells in response to specific growth factors. Bone.

[78] Setzu A., Lathia J.D., Zhao C., Wells K., Rao M.S., Ffrench-Constant C., Franklin R.J.M. (2006). Inflammation stimulates myelination by transplanted oligodendrocyte precursor cells. Glia.

[79] McCulloch K., Litherland G.J., Rai T.S. (2017). Cellular senescence in osteoarthritis pathology. Aging Cell.

[80] Brandl A., Hartmann A., Bechmann V., Graf B., Nerlich M., Angele P. (2011). Oxidative stress induces senescence in chondrocytes. J. Orthop. Res..

[81] Rim Y.A., Nam Y., Ju J.H. (2020). The Role of Chondrocyte Hypertrophy and Senescence in Osteoarthritis Initiation and Progression. Int. J. Mol. Sci..

[82] Vinatier C., Dominguez E., Guicheux J., Caramés B. (2018). Role of the Inflammation-Autophagy-Senescence Integrative Network in Osteoarthritis. Front. Physiol..

